# Supplementary material for: Air quality and obesity at older ages in China: The role of duration, severity and pollutants
Source: PLoS One. 2019 Dec 11;14(12):e0226279. doi: 10.1371/journal.pone.0226279 (PMC6905559; doi:10.1371/journal.pone.0226279)
Supplement: S1 Table — (DOCX) [file pone.0226279.s001.docx]

**S1 Table. Marginal impacts of average AQI on general obesity and central obesity among older people from the China Health and Retirement Longitudinal Study 2015**

|  | **General obesity** | | | **Abdominal obesity** | | |
| --- | --- | --- | --- | --- | --- | --- |
| **Air pollution** | dy/dx | 95%CI | p-value | dy/dx | 95%CI | p-value |
| **Average AQI**  (Standardised) | 0.028^***^ | (0.017, 0.039) | P<0.0001 | 0.062^***^ | (0.044, 0.080) | P<0.0001 |

AQI, Air quality index; GDP, Gross Domestic Product; CI, confidence interval.

Fully adjusted pollutants, age, gender, education, log transformation of household consumption per capita, *Hukou*, place of residence, region, log transformation of local GDP per capita, manufacturing share of GDP, number of hospital beds per 1000 and disability (not reported here).

*p<0.05, ** p<0.01, ***p<0.001
