# Supplementary material for: Air quality and obesity at older ages in China: The role of duration, severity and pollutants
Source: PLoS One. 2019 Dec 11;14(12):e0226279. doi: 10.1371/journal.pone.0226279 (PMC6905559; doi:10.1371/journal.pone.0226279)
Supplement: S2 Table — (DOCX) [file pone.0226279.s002.docx]

S2 Table. Regression results (Odds ratio and 95%CI) for testing mediating role of physical activity by further adjusting for physical activity

|  | **General obesity** | |  | **Abdominal obesity** | |
| --- | --- | --- | --- | --- | --- |
| **Variables** | OR | 95%CI |  | OR | 95%CI |
| **Intercept** | 0.008 | (0.0001, 1.077) |  | 0.777 | (0.041, 14.764) |
| **Average AQI** (Standardised) | 1.394^***^ | (1.170, 1.662) |  | 1.466^***^ | (1.296, 1.659) |
| **Age** (Centred at 60 years) | 0.956^**^ | (0.929, 0.983) |  | 0.990 | (0.973, 1.007) |
| **Sex** (Ref: Male) | 1.820^***^ | (1.310, 2.528) |  | 6.152^***^ | (4.653, 8.134) |
| **Marital status** (Ref: No) | 0.934 | (0.616, 1.417) |  | 1.331 | (0.997, 1.777) |
| **Education** (Ref: Illiterate) |  |  |  |  |  |
| Primary or secondary | 0.928 | (0.647, 1.331) |  | 1.087 | (0.843, 1.401) |
| Tertiary | 0.813 | (0.40, 1.651) |  | 1.170 | (0.7126, 1.884) |
| **Household consumption per capita** (Logged) | 1.101 | (0.931, 1.301) |  | 1.166^**^ | (1.044, 1.302) |
| ***Hukou*** (Ref: Urban) | 0.810 | (0.514, 1.278) |  | 0.717^*^ | (0.526, 0.978) |
| **Place of residence** (Ref: Urban) | 0.761 | (0.509, 1.138) |  | 0.681^**^ | (0.527, 0.881) |
| **Local GDP per capita** (Logged) | 1.107 | (0.636, 1.929) |  | 0.735 | (0.525, 1.028) |
| **Manufacturing share of GDP, %** | 1.010 | (0.984, 1.037) |  | 1.003 | (0.987, 1.020) |
| **Number of hospital beds per 1000** | 1.027 | (0.868, 1.216) |  | 1.240^***^ | (1.109, 1.386) |
| **Disability** (Ref: No difficulty) | 1.994^***^ | (1.431, 2.779) |  | 1.763^***^ | (1.377, 2.256) |
| **Physical inactivity** (Ref: Active) | 1.231 | (0.904, 1.676) |  | 1.204 | (0.974, 1.489) |
| **N** | 2121 |  |  | 2136 |  |

AQI, Air quality index; GDP, Gross Domestic Product; OR, odds ratio; CI, confidence interval; Additionally adjusted for region (not reported).

*p<0.05, ** p<0.01, ***p<0.001
