# Supplementary material for: Air quality and obesity at older ages in China: The role of duration, severity and pollutants
Source: PLoS One. 2019 Dec 11;14(12):e0226279. doi: 10.1371/journal.pone.0226279 (PMC6905559; doi:10.1371/journal.pone.0226279)
Supplement: S3 Table — (DOCX) [file pone.0226279.s003.docx]

**S3 Table. Regression results (Odds ratio and 95%CI) for associations between air quality measures and physical inactivity among older people from the China Health and Retirement Longitudinal Study 2015**

|  | **Physical inactivity** | |
| --- | --- | --- |
| **Air pollution** | OR | 95%CI |
| **Intercept** | 0.868 | (0.028, 26.769) |
| **Average AQI** (Standardized) | 1.190^**^ | (1.042, 1.359) |
| **Age** (Centred at 60 years) | 1.086^***^ | (1.065, 1.108) |
| **Sex** (Ref: Male) | 1.166 | (0.934, 1.455) |
| **Marital status** (Ref: No) | 0.938 | (0.689, 1.277) |
| **Education** (Ref: Illiterate) |  |  |
| Primary or secondary | 0.887 | (0.681, 1.157) |
| Tertiary | 0.799 | (0.493, 1.298) |
| **Household consumption per capita** (Logged) | 0.860^*^ | (0.761, 0.972) |
| ***Hukou*** (Ref: Urban) | 0.882 | (0.630, 1.234) |
| **Place of residence** (Ref: Urban) | 0.661^**^ | (0.488, 0.894) |
| **Local GDP per capita** (Logged) | 1.077 | (0.730, 1.588) |
| **Manufacturing share of GDP, %** | 0.991 | (0.973, 1.009) |
| **Number of hospital beds per 1000** | 0.938 | (0.828, 1.062) |
| **Disability** (Ref: No difficulty) | 1.623^***^ | (1.259, 2.093) |

AQI, Air quality index; GDP, Gross Domestic Product; OR, odds ratio; CI, confidence interval.

Fully adjusted pollutants, age, gender, education, log transformation of household consumption per capita, *Hukou*, place of residence, region, log transformation of local GDP per capita, manufacturing share of GDP, number of hospital beds per 1000 and disability (not reported here).

*p<0.05, ** p<0.01, *** p<0.001
