# Supplementary material for: Air quality and obesity at older ages in China: The role of duration, severity and pollutants
Source: PLoS One. 2019 Dec 11;14(12):e0226279. doi: 10.1371/journal.pone.0226279 (PMC6905559; doi:10.1371/journal.pone.0226279)
Supplement: S1 Fig — Predicted general obesity (Left) and abdominal obesity (Right) among older adults by disability, the China Health and Retirement Longitudinal Study 2015. (DOCX) [file pone.0226279.s004.docx]

**S1 Fig**. **Predicted general obesity (Left) and abdominal obesity (Right) among older adults by disability, the China Health and Retirement Longitudinal Study 2015**
