# Supplementary material for: Air quality and obesity at older ages in China: The role of duration, severity and pollutants
Source: PLoS One. 2019 Dec 11;14(12):e0226279. doi: 10.1371/journal.pone.0226279 (PMC6905559; doi:10.1371/journal.pone.0226279)
Supplement: Attachment — Submitted filename: Response to reviewers.docx [file pone.0226279.s005.docx]

PONE-D-19-21831

Exposure to ambient air pollution and obesity at older ages in China: the role of duration, severity and pollutants

PLOS ONE

Dear Dr Zhang,

To enhance the reproducibility of your results, we recommend that if applicable you deposit your laboratory protocols in [protocols.io](http://protocols.io/), where a protocol can be assigned its own identifier (DOI) such that it can be cited independently in the future. For instructions see: <http://journals.plos.org/plosone/s/submission-guidelines#loc-laboratory-protocols>

Please include the following items when submitting your revised manuscript:

8. A rebuttal letter that responds to each point raised by the academic editor and reviewer(s). This letter should be uploaded as separate file and labeled 'Response to Reviewers'.
9. A marked-up copy of your manuscript that highlights changes made to the original version. This file should be uploaded as separate file and labeled 'Revised Manuscript with Track Changes'.
10. An unmarked version of your revised paper without tracked changes. This file should be uploaded as separate file and labeled 'Manuscript'.

**Journal Requirements:**

1. When submitting your revision, we need you to address these additional requirements.

Please ensure that your manuscript meets PLOS ONE's style requirements, including those for file naming. The PLOS ONE style templates can be found at

<http://www.journals.plos.org/plosone/s/file?id=wjVg/PLOSOne_formatting_sample_main_body.pdf> and <http://www.journals.plos.org/plosone/s/file?id=ba62/PLOSOne_formatting_sample_title_authors_affiliations.pdf>

**Thank you. We have formmated the manuscript according to the requirements.**

2. Thank you for stating the following financial disclosure: “No”   a) Please provide an amended Funding Statement that declares *all* the funding or sources of support received during this specific study (whether external or internal to your organization) as detailed online in our guide for authors at <http://journals.plos.org/plosone/s/submit-now>.     b)  Please state what role the funders took in the study.  If any authors received a salary from any of your funders, please state which authors and which funder. If the funders had no role, please state: "The funders had no role in study design, data collection and analysis, decision to publish, or preparation of the manuscript."

c) If the study was unfunded, please state "The author(s) received no specific funding for this work."

Please include your amended statements within your cover letter; we will change the online submission form on your behalf.

**Thank you. We amended funding statements and included it within the cover letter.**

3. We note that  Figure(s) 2,3,& 4 in your submission contain [map/satellite] images which may be copyrighted. All PLOS content is published under the Creative Commons Attribution License (CC BY 4.0), which means that the manuscript, images, and Supporting Information files will be freely available online, and any third party is permitted to access, download, copy, distribute, and use these materials in any way, even commercially, with proper attribution. For these reasons, we cannot publish previously copyrighted maps or satellite images created using proprietary data, such as Google software (Google Maps, Street View, and Earth). For more information, see our copyright guidelines: <http://journals.plos.org/plosone/s/licenses-and-copyright>.

**Thank you very much.**

**The figures 2-4 were produced by authors through the software of ArcMap 10.4. The shapefile of China’s municipal boundaries (polygons) are accessed from the datasets of the National Geomatics Center of China (**[**http://www.ngcc.cn/ngcc/**](http://www.ngcc.cn/ngcc/)**). Therefore, we own the copyrights. They are free to download by an application. We merged corresponding municipal boundaries to obtain seven regions of Southwest China, Northwest China, Northeast China, Central China, North China, East China, South China according to the regional division of China’s national official statistical yearbook. Consequently, we mapped the polygons of municipalities with different colors with the attributes their air quality index, exposures to PM2.5 and exposures to PM10 acquired from the real-time data of China National Environmental Monitoring Center (**[**http://www.cnemc.cn**](http://www.cnemc.cn/)**). The website publishes daily air quality data and major pollutants of Chinese municipalities in the past years.**

Reviewer #1: Reviewer comments for manuscript PONE-D-19-21831

The authors present findings from their study exploring the association of general and pollutant-specific air quality with general and central obesity, using data from CHARLS study. Although the manuscript is generally well-written, there are methodological issues which need to be addressed to improve the manuscript.

**Thank you for your comments. We have addressed the methodological issues that you kindly raised and please see our responses and an updated version.**

1. Adapt the title to reflect air quality index as the exposure metric

**Thank you. We have modifited the title ‘Air quality and obesity at older ages in China: The role of duration, severity and pollutants’.**

2. The study included 4364 out of 9069 eligible participants (48%). The authors should there present a table likely the current Table 1, where they summarize all the covariates between the included (4364) and the excluded (4732) CHARLS participants, and report the p-values of the differences in each covariate between the groups. You could then discuss the generalizability of This should replace the current Table 1 as it is not so informative on the research question.

**Thank you very much. We replaced the orginal table with the following table. We have updated results according to the updated table and further discussed the influences on our findings in the discussion.**

**After removing those who had missing data on obesity outcomes (n=1,787), the eligible older adults dropped to 7,309. Our analysis was based on 4364 out of 7,309. The table below does show that there are some important differences between the included and excluded sample that may bias our estimates and may comprise the generalizability of our findings, however, in many cases the differences are small in size. The exceptions to this are: gender, where the excluded sample has a greater proportion of women; marital status, where the excluded sample is more likely to be single; disability, where the excluded sample is more likely to have a limitation; physical activity, where the excluded sample is less likely to be inactive. Importantly, these factors are all included in our models, which gives more confidence in the conclusions drawn.**

Table 1. Sample description of included and exluded older people from the China Health and Retirement Longitudinal Study 2015

|  | **Included sample** | | **Excluded sample** | | **Difference** |
| --- | --- | --- | --- | --- | --- |
| **Variables** | N | Mean (sd)/Percentage | N | Mean (sd)/Percentage | *P-value* |
| **Outcomes** |  |  |  |  |  |
| General obesity | 4364 | 11.16% | 2945 | 10.12% | 0.159 |
| Abdominal obesity | 4359 | 54.85% | 2937 | 51.75% | <0.01 |
| **Age** (years) | 4364 | 67.53 (6.11) | 2929 | 69.32 (7.50) | <0.001 |
| **Gender** |  |  |  |  | <0.001 |
| Male | 2253 | 51.63% | 1366 | 46.38% |  |
| Female | 2111 | 48.37% | 1579 | 53.62% |  |
| **Marital status** |  |  |  |  | <0.001 |
| Not married or partnered | 684 | 15.67% | 806 | 27.38% |  |
| Married or partnered | 3680 | 84.33% | 2138 | 72.62% |  |
| **Education** |  |  |  |  | <0.001 |
| Illiterate | 1382 | 31.67% | 1154 | 39.24% |  |
| Primary or secondary | 2654 | 60.82% | 1632 | 55.49% |  |
| Tertiary | 328 | 7.52% | 155 | 5.27% |  |
| **Household consumption per capita** (Logged) (RMB) | 4364 | 8.97 (0.99) | 341 | 8.94 (1.04) | 0.6220 |
| **Hukou** |  |  |  |  | <0.001 |
| Urban Hukou | 1065 | 24.40% | 432 | 17.50% |  |
| Rural Hukou | 3299 | 75.60% | 2037 | 82.50% |  |
| **Place of residence** (Ref: Urban) |  |  |  |  | <0.05 |
| Urban communities | 1729 | 39.62% | 1098 | 37.28% |  |
| Rural communities | 2635 | 60.38% | 1847 | 62.72% |  |
| **Disability** |  |  |  |  | <0.001 |
| No difficulty | 3303 | 75.69% | 2064 | 70.49% |  |
| At least one difficulty | 1061 | 24.31% | 864 | 29.51% |  |
| **Physical inactivity** |  |  |  |  | <0.001 |
| Active | 1282 | 60.44% | 738 | 54.71% |  |
| Inactive | 839 | 39.56% | 611 | 45.29% |  |
| **GDP per capita** (Logged) (RMB) | 4364 | 10.58 (0.53) | 2945 | 10.62 (0.54) | <0.01 |
| **Manufacturing share of GDP, %** | 4364 | 45.99 (7.85) | 2945 | 45.81 (7.72) | 0.3373 |
| **Number of hospital beds per 1000** | 4364 | 4.67 (1.50) | 2945 | 4.69 (1.65) | 0.4612 |
| **Air quality** |  |  |  |  |  |
| Average AQI | 4364 | 88.73(23.14) | 2945 | 85.72(23.97) | <0.001 |
| No. of days AQI ≥ 101 | 4364 | 101(64) | 2945 | 94(66) | <0.001 |
| No. of days AQI ≥ 201 | 4364 | 15(16) | 2945 | 13(16) | <0.001 |
| No. of days AQI ≥ 301 | 4364 | 3(6) | 2945 | 3(5) | <0.01 |
| ***No. of days exposed to*** |  |  |  |  |  |
| SO2 | 4364 | 4(10) | 2945 | 4(10) | 0.5704 |
| NO2 | 4364 | 9(16) | 2945 | 11(20) | <0.001 |
| PM2.5 | 4364 | 144(60) | 2945 | 136(58) | <0.001 |
| PM10 | 4364 | 76(41) | 2945 | 76(41) | <0.001 |
| O3 | 4364 | 71(39) | 2945 | 71(39) | 0.7684 |
| CO | 4364 | 3(8) | 2945 | 3(8) | <0.01 |

AQI, Air quality index; GDP, Gross Domestic Product

3. Air quality indicators were assigned on city level. How many city-level clusters did the study include? How did you deal with clustering in AQI in the analyses? I suggest sensitivity analyses where city-level is considered as fixed as well as random effect, especially for the standardized continuous AQI models

**Thank you very much. Air quality data of 118 cities were employed in the analyses. However, city-level variance was not taken into account, as it was small and non-significant compared to individual-, household- and community-levels. Moreover, we chose three-level instead of four-level (further adding city-level) multinomial logistic regressions because the latter failed to converage due to complex model structures.**

**We have made changes to 2.6 Analytical strategy.**

4. It is unclear in the methodology, the definition of the overall AQI. This was mentioned in the limitations, but should be moved to the methodology. How was the standardization of the AQI done?

**Thank you very much. We have modified the term ‘overall AQI’ to average AQI.**

**The annual average AQI is calculated based on the daily concentrations of the six criteria pollutants, namely** [**sulfur dioxide**](https://en.wikipedia.org/wiki/Sulfur_dioxide) **(SO2),** [**nitrogen dioxide**](https://en.wikipedia.org/wiki/Nitrogen_dioxide) **(NO2), suspended particulates smaller than 10 μm in** [**aerodynamic diameter**](https://en.wikipedia.org/wiki/Aerodynamic_diameter) **(PM10), suspended particulates smaller than 2.5 μm in** [**aerodynamic diameter**](https://en.wikipedia.org/wiki/Aerodynamic_diameter) **(PM2.5) (****Ministry of Ecology and Environment of the People's Republic of China 2012a****)** [**carbon monoxide**](https://en.wikipedia.org/wiki/Carbon_monoxide) **(CO), and** [**ozone**](https://en.wikipedia.org/wiki/Ozone) **(O3) measured at the monitoring stations across each city (****Ministry of Ecology and Environment of the People's Republic of China 2012b****). The overall AQI ranges 0-500.**

**AQI was standardized by substracting off the mean and dividing by the standard deviation. We have made changes to the manuscript.**

5. What is the correlation of physical inactivity and disability? I expect this to be very high, given that the disability should hinder physical activity, and the finding of a strong association between disability and physical inactivity showed in Table A3. I suggest doing the effect modification and mediation analysis on both physical activity and disability, limited to those who reported physical activity. If the correlation is very high, you may then consider combining both into one variable and do the mediation/effect modification in the entire 4364 sample.

**Thank you very much.**

**We found correlation between physical inactivity and disability is 0.1329 in our study, which is quite low. We believe it might be partly due to how these two variables were measured in this study. Disability is an indicator of physical functioning which was based on activities of daily living (ADL) dressing, bathing, eating, getting into or out of bed, toilet, controlling urination or defecation, and instrumental ADLs (IADLs): household chores, cooking, shopping, managing money and taking medication. If participants have difficulty in one of the above activities, then he/she is considered living with disability. However, physical inactivity was a dichotomous variable with 0 indicating respondent taking part in vigorous or moderate physical activities for at least 10 minutes every week and 1 representing no or light activities. This is not an ideal measure as it was only asked for a subsample of eligible respondents and, more importantly, it is oversimplified that did not adequately capture the duration and intensity of weight-related activity. We have reported this in Discussion.**

**In addition, we chose to do mediation analysis with physical inactivity because we think it is in the causal pathway from air pollution to obesity. The main reason for the interaction term with disability is because air pollution effect of obesity may be moderated by levels of physical functioning.**

6. Table A2: Include the AQI estimates from Tables 2 and 3 for direct comparison, instead of just referring to them in the title.

**Thank you very much. We have modified the title of the Table A2. Since Tables 2 and 3 mainly focus on main effects of PM2.5 and obesity outcomes and the interaction with disability. The tables may seem confusing. Therefore, we decide to keep two tables separate.**

7. Consider reporting the effect estimates in 2 decimal places instead of 3. Looks cleaner.

**Thank you very much. Because the magnitudes of some of our estimates are quite small and the changes in magnitudes may become invisible if we only report 2 decimials. Therefore, in order to show the precise effect size changes, we decided to keep 3 decimals.**

8. Manuscript contains some typographical errors that should be corrected

Reviewer #2: This study looks at the association between air pollution (AQI) and obesity in a cohort of Chinese elderly using a cross-sectional design. The detailed cohort characteristics and geographic variety of the participants are major strengths of the study, but the reliance on AQI for exposure assessment is a weakness. Overall the paper is well-written, and obesity (and central obesity) is an interesting outcome to evaluate given the relative lack of evidence in the current literature. I suggest several additions to strengthen the paper.

**Thank you very much. AQI is an overall measure of air quality based on the six criteria pollutants, namely** [**sulfur dioxide**](https://en.wikipedia.org/wiki/Sulfur_dioxide) **(SO2),** [**nitrogen dioxide**](https://en.wikipedia.org/wiki/Nitrogen_dioxide) **(NO2), suspended particulates smaller than 10 μm in** [**aerodynamic diameter**](https://en.wikipedia.org/wiki/Aerodynamic_diameter) **(PM10), suspended particulates smaller than 2.5 μm in** [**aerodynamic diameter**](https://en.wikipedia.org/wiki/Aerodynamic_diameter) **(PM2.5)** [**carbon monoxide**](https://en.wikipedia.org/wiki/Carbon_monoxide) **(CO), and** [**ozone**](https://en.wikipedia.org/wiki/Ozone) **(O3). We have highlighted the limitation of using AQI in the discussion.**

1. Consider adding to discussion as to why associations with particulate matter but not the other pollutants were observed, e.g. are there different potential mechanisms involved?

**Thank you very much.**

**Also, we found that chronic exposures to PM2.5 and PM10 play a significant part in the adverse impact of air pollution on obesity outcomes among older Chinese adults. On one hand, air pollution, such as PM, are known to increase infiltration and activation of immune-competent cells (i.e., monocytes and macrophages) in adipose and other tissues (****Zou 2010****). On the other, epidemiological evidence suggests that obesity may increase susceptibility to the impacts of PM2.5 on the cardiovascular conditions (****Weichenthal *et al.* 2014****). For example, obesity may enhance the effects of air pollution on the CVDs and stroke among Chinese adults in particular among women (****Qin *et al.* 2015****). We did not find significant impacts of other pollutants such as CO, NO2, SO2 and O3 on obesity outcomes at older ages. One of the possible explanations may be due to the fact that the durations of exposure to these pollutants are shorter than PM in China. Our estimates thus may be underpowered. Moreover, there is little evidence suggesting whether and how certain pollutants such as NO2, SO2 and O3 may be held accountable for obesity (****Dong *et al.* 2014****) and it remains unknown for older people.**

2. Introduction is rather long and could be shorter and more focused. Please consider combining paragraphs 1 and first half of paragraph 2, and shortening other paragraphs.

**Thank you very much. We have modified the introduction section and shorten some parts. Please see the updated version.**

3. It’s a bit unclear about how the individual pollutants are modeled in section 2.4. What do you mean exactly by “days of exposure to major pollutants?” Same approach as AQI?

**Thank you very much. Yes, the approach was the same as AQI.**

**Environment Quality Report provided the most important pollutant every day among six pollutants including PM2.5, PM10, CO, NO2, SO2 and O3, we calculate how many days of exposure to the major pollutants within one year. We have made changes and please see the updated version.**

4. Only a percentage of the entire cohort was included in the study. Are there significant differences in cohort characteristics between the excluded participants and included participants in the study?

**Thank you very much. We replaced the orginal table with the new table and also reported the p-values of the differences in each covariate between the groups. Please refer to Table 1. We have updated results according to the updated table and further discussed the influences on our findings in the discussion.**

Minor Comments

1. Please state population size in the abstract.

**Thank you, we have modified it.**

2. Clarify the cross-sectional nature of the study design in the methods.

**Thank you, we have modified it.**

3. Clarify “standardized AQI” in section 2.4.

**Thank you, we have modified it. AQI was standardized by substracting off the mean and dividing by the standard deviation.**

4. Consider restating “older Chinese with disability” to “elderly Chinese with disability” when discussing vulnerable subpopulations. Older Chinese sounds like age is also a modifier.

**Thank you, we have modified it.**

6. PLOS authors have the option to publish the peer review history of their article ([what does this mean?](https://journals.plos.org/plosone/s/editorial-and-peer-review-process#loc-peer-review-history)). If published, this will include your full peer review and any attached files.   If you choose “no”, your identity will remain anonymous but your review may still be made public.   **Do you want your identity to be public for this peer review?** For information about this choice, including consent withdrawal, please see our [Privacy Policy](https://www.plos.org/privacy-policy).

Reviewer #1: No

Reviewer #2: No

[NOTE: If reviewer comments were submitted as an attachment file, they will be attached to this email and accessible via the submission site. Please log into your account, locate the manuscript record, and check for the action link "View Attachments". If this link does not appear, there are no attachment files to be viewed.]

While revising your submission, please upload your figure files to the Preflight Analysis and Conversion Engine (PACE) digital diagnostic tool, <https://pacev2.apexcovantage.com/>. PACE helps ensure that figures meet PLOS requirements. To use PACE, you must first register as a user. Registration is free. Then, login and navigate to the UPLOAD tab, where you will find detailed instructions on how to use the tool. If you encounter any issues or have any questions when using PACE, please email us at [figures@plos.org](mailto:figures@plos.org). Please note that Supporting Information files do not need this step.

**References**

Dong, G. H., Qian, Z., Liu, M. M., Wang, D., Ren, W. H., Flick, L. H., Fu, J., Wang, J., Chen, W., Simckes, M. and Trevathan, E. 2014. Ambient air pollution and the prevalence of obesity in Chinese children: The seven northeastern cities study. *Obesity,* 22**,** 795-800.

Ministry of Ecology and Environment of the People's Republic of China 2012a. Ambient Air Quality Standards "环境空气质量标准" Ministry of Ecology and Environment of the People's Republic of China.

Ministry of Ecology and Environment of the People's Republic of China 2012b. Technical Regulation on Ambient Air Quality Index (on trial) "环境空气质量指数（AQI) 技术规定（试行)”. Ministry of Ecology and Environment of the People's Republic of China.

Qin, X. D., Qian, Z., Vaughn, M. G., Trevathan, E., Emo, B., Paul, G., Ren, W. H., Hao, Y. T. and Dong, G. H. 2015. Gender-specific differences of interaction between obesity and air pollution on stroke and cardiovascular diseases in Chinese adults from a high pollution range area: A large population based cross sectional study. *Sci Total Environ,* 529**,** 243-8.

Weichenthal, S., Hoppin, J. A. and Reeves, F. 2014. Obesity and the cardiovascular health effects of fine particulate air pollution. *Obesity,* 22**,** 1580-9.

Zou, M. H. 2010. Is NAD (P) H oxidase a missing link for air pollution-enhanced obesity? *Arteriosclerosis, Thrombosis, and Vascular Biology,* 30**,** 2323-2324.
